# Epidemiological Analysis of Environmental Factors Affecting Porcine Pleuropneumonia in a Herd Endemic for *Actinobacillus pleuropneumoniae*

**DOI:** 10.3390/pathogens15050513

**Published:** 2026-05-11

**Authors:** Iulia Barna, Ion Tașca, Adriana-Iuliana Toader, Andrei Ungur

**Affiliations:** Department of Porcine Health Management, Faculty of Veterinary Medicine, University of Agricultural Sciences and Veterinary Medicine of Cluj-Napoca, 400372 Cluj-Napoca, Romania; ion.tasca@usamvcluj.ro (I.T.); adriana-iuliana.toader@student.usamvcluj.ro (A.-I.T.); andrei.ungur@usamvcluj.ro (A.U.)

**Keywords:** *Actinobacillus pleuropneumoniae*, swine pleuropneumonia, climatic factors

## Abstract

*Actinobacillus pleuropneumoniae* (APP) is the bacterial agent associated with porcine pleuropneumonia, a severe respiratory disease with significant economic impact due to increased mortality and elevated therapeutic costs, whose progression may be exacerbated by environmental factors. This study evaluates the impact of climatic factors, particularly temperature and relative humidity, on mortality in finishing pigs during the fattening phase within a herd with endemic porcine pleuropneumonia. Data were collected from a commercial swine farm between January 2021 and December 2024, including records of local climatic conditions (daily average temperature and relative humidity) and pig mortality rates. Statistical analysis revealed a positive correlation between periods of elevated temperature or humidity and increased mortality associated with respiratory diseases, suggesting that climatic stress contributes to higher mortality in this herd with endemic APP.

## 1. Introduction

*Actinobacillus pleuropneumoniae* (APP) causes porcine pleuropneumonia, a highly contagious respiratory disease associated with significant economic losses [1]. The bacterial cells are Gram-negative, non-flagellated, asporogenous, with a morphological variability ranging from coccoid to rod-shaped forms, typically found singly, in pairs, or arranged in short chains [2].

APP displays considerable serotype diversity, comprising 19 distinct serotypes [3], primarily differentiated by variations in the capsule synthesis genes, as well as structural differences in capsular polysaccharides and cell wall lipopolysaccharides [3,4]. Temporal and regional variation in serotype prevalence has been observed, with clinical pig isolates in Europe currently dominated by serotypes 9, 2, and 7, reflecting the dynamic distribution of the pathogen [5]. Recent European surveillance data highlight notable regional differences: in Hungary, serotypes 2 and 13 currently predominate [6]; in Italy, serotypes 9/11 and 2 are most frequent, with additional reports of serotypes 2 and 6 [7,8]; historical data from Belgium indicate serotypes 2, 3, and 9 as the most commonly isolated strains [9]. Switzerland displays a distinctive profile, with serotype 2 considered eradicated [10], while in England and Wales, serotype 8 predominates and serotypes 2, 6, 7, and 12 occur at lower frequencies [11].

This variability in virulence and geographic distribution poses significant challenges for effective control strategies and vaccine formulation [12].

Direct transmission of APP between pigs is regarded as the principal pathway for disease dissemination, given the bacterium’s limited environmental survival [13]. This bacterium is capable of inducing lung lesions independently of co-infection with other bacteria or viruses, although its pathogenicity is frequently exacerbated by physical and environmental stressors [14]. The pathogenesis of pleuropneumonia involves three fundamental stages: initial colonization of the host’s cells, interference with the mucociliary and immune clearance mechanisms and compromise of tissue integrity [12].

Porcine pleuropneumonia may manifest in acute, chronic or subclinical forms, with the acute form often characterized by anorexia, fever, respiratory distress, and, in some cases, sudden death [15]. The predominant gross pathological lesions associated with this disease are hemorrhagic necrotizing pneumonia and fibrinous pleuritis [16]. Post-mortem evaluation can be performed using standardized lung lesion scoring systems for APP [16].

Factors such as overcrowding, poor ventilation and abrupt climatic changes—particularly fluctuations in temperature and high relative humidity—facilitate the development and transmission of the disease, contributing to increased morbidity and mortality [17].

This study analyzes the influence of climatic factors—particularly temperature, humidity, and ventilation—on the monthly mortality associated with porcine pleuropneumonia in a commercial farm. Its aim is to identify correlations between these parameters and disease outbreaks, in order to detect high-risk periods and optimize preventive measures. The findings may contribute to improving microclimate management and adjusting preventive strategies according to seasonal variations. By highlighting the impact of meteorological conditions on the progression of pleuropneumonia, the present study provides a practical approach that may help reduce disease incidence, improve farm economic efficiency, and enhance herd health.

## 2. Materials and Methods

### 2.1. Farm Description

The present study was conducted on a commercial swine finishing farm located in the village of Cimișeni, Criuleni District, Moldova, where climatic factors vary significantly between seasons. The analysis spanned a continuous four-year timeframe, from January 2021 to December 2024, comprising a total of 48 consecutive months. The potential influence of meteorological parameters—monthly average temperature, monthly precipitation and relative humidity—on the monthly mortality rate of finishing pigs during the fattening period was investigated under conditions of endemic circulation of APP.

Porcine pleuropneumonia represents a major health concern within the swine herd of this farm, with a significant impact on animal productivity and welfare. The farm has 3500 sows, 1500 weaned piglets and 10,000 finishing pigs in a “farrow-to-finish” production system and has experienced a persistent issue with respiratory diseases in finishing pigs.

Clinical signs ranged from sudden death to chronic pneumonia, accompanied by weight loss and slow growth. The animals exhibited lethargy around days 95–105, were reluctant to stand and drink water and refused to feed. They showed severe respiratory clinical signs, including dyspnea, coughing, and open-mouth breathing. Nasal discharge and hyperthermia (40.5–41 °C) were also observed.

The overall mortality rate among finishing pigs on the farm ranged from 5% to 18%, while mortality due to respiratory diseases varied between 4% and 16% per week during the same period.

The farm had a history of infections with pathogens like PRRSV genotype 1, *Mycoplasma hyopneumoniae*, *Suid herpesvirus 1* (the causative agent of Aujeszky’s disease) and *swine influenza virus*. These infectious agents are currently kept under control through vaccination programs implemented on the farm.

The study was conducted in a farm equipped with a conventional ventilation system, which operates without advanced automated controls and is therefore directly influenced by outdoor weather conditions.

Farm management practices remained largely consistent throughout the four-year study period with regard to nutrition and biosecurity protocols. However, variations were implemented in ventilation settings and therapeutic strategies targeting APP. Ventilation parameters, including temperature and airflow rates, were periodically adjusted in an attempt to mitigate disease outbreaks, although without consistent success. Pigs were transferred to the fattening units at approximately 23 °C, with a gradual decrease to 19 °C over a period of 6–8 weeks, depending on the season. Housing conditions consisted of partially slatted flooring (30%) and solid flooring (70%), which contributed to slightly increased indoor humidity during winter, particularly under conditions of external fog or low atmospheric pressure.

Multiple antimicrobial protocols were applied across different groups, including water-administered treatments (florfenicol, amoxicillin, tilmicosin, tiamulin combined with doxycycline and florfenicol combined with amoxicillin), often supplemented with paracetamol, as well as injectable therapies (marbofloxacin, enrofloxacin, amoxicillin–clavulanic acid and florfenicol).

The general vaccination scheme was consistently applied across the study period for both sows and pigs. Sows were routinely vaccinated four times per year against porcine reproductive and respiratory syndrome (PRRS) and Aujeszky’s disease, and twice per year against swine influenza.

Pigs followed a standard vaccination schedule that included immunization against edema disease at 7 days of age, *Mycoplasma hyopneumoniae* at 14 days and a combined vaccine against porcine circovirus type 2 (PCV2) and PRRS at 21 days of age. Vaccination against Aujeszky’s disease was administered at approximately 70 days of age, with a booster at around 100 days. In some instances, the Aujeszky’s disease vaccine was combined with vaccination against APP.

Several vaccination protocols targeting APP were implemented during the study period. One approach involved the use of a commercial vaccine (*Porcilis APP*, MSD Animal Health, Boxmeer, The Netherlands), administered as a two-dose regimen at 42 and 70 days of age; in some cases, a third dose was administered at approximately 100 days. An alternative strategy consisted of post-outbreak vaccination, in which pigs were first treated with antimicrobial therapy during acute disease cases, followed by APP vaccination seven days later. This approach yielded limited efficacy.

Additional vaccination protocols included the use of another commercially available APP vaccine (*Coglapix*, Ceva Santé Animale, Libourne, France) administered at 49 and 70 days of age, or autogenous vaccines (Exopol, Zaragoza, Spain) administered at 45 and 65 days of age. Despite these varied strategies, the overall effectiveness of APP vaccination protocols remained inconsistent.

Diagnostic monitoring was performed regularly, with polymerase chain reaction (PCR) testing (EXOone, Exopol, Zaragoza, Spain) conducted approximately every 4–6 months. Samples for PCR analysis consisted of pulmonary swabs collected both in transport medium (Amies transport medium, Copan Italia S.p.A., Brescia, Italy) and as dry swabs. These samples were subsequently subjected to bacteriological culture, followed by serotyping of the isolated strains. Furthermore, necropsies were performed on approximately 90% of deceased animals to assess pathological changes and confirm the presence of APP-associated lesions. Overall mortality in growing and finishing pigs averaged approximately 8% within a production system of around 110,000 pigs per year.

### 2.2. Source of Data

The data originate from the veterinary health records of the farm located in the village of Cimișeni, Criuleni District, Moldova. The period covered by the epidemiological study of environmental factors influencing the monthly mortality associated with porcine pleuropneumonia caused by APP is 2021–2024.

For each month, data were collected and compiled into a table that included the following variables: year, month (in both text and numeric formats), season (classified as winter, spring, summer, and autumn), average temperature (expressed in degrees Celsius), average precipitation (mm), average relative humidity (%), and pig mortality rate (%).

Meteorological data were retrieved from the AccuWeather (AccuWeather, Inc., State College, PA, USA) official database corresponding to the study area, while monthly mortality information was provided by the veterinarian responsible for the sanitary supervision of the herd. The epidemiological parameters collected include mortality, monthly incidence, and the current vaccination protocol.

### 2.3. Analytical Methods

For statistical analysis, the data were initially processed in Microsoft Excel (Microsoft Corporation, Redmond, WA, USA) and subsequently imported into Python (version 3.12.13; Python Software Foundation, Wilmington, DE, USA) environments using the pandas (version 2.2.2), matplotlib (version 3.10.0), seaborn (version 0.13.2), and stats models (version 0.14.6) libraries. The data structure allowed for an integrative approach, including: descriptive statistical analysis (mean, minimum, maximum, standard deviation), Pearson linear correlation analysis between mortality and climatic variables, seasonality analysis by comparing average mortality across seasons, multiple linear regression modeling to estimate the combined influence of climatic variables on mortality, time series modeling using a Seasonal Autoregressive Integrated Moving Average (SARIMA) model to evaluate seasonal trends and make forecasts for upcoming periods. This multifactorial approach enabled the identification of recurring patterns and the estimation of the relative importance of each climatic parameter within the context of biosecurity and veterinary sanitary management of swine farms.

## 3. Results

The results obtained regarding mortality associated with cases of porcine pleuropneumonia were analyzed according to season. The data were organized chronologically by years and seasons (spring, summer, autumn, winter), highlighting monthly variations.

### 3.1. Data for 2021

The results obtained for the year 2021 indicate significant variations in mortality depending on the season, correlated with the recorded meteorological parameters.

During the winter months, mortality ranged between 2.13% (February) and 2.42% (December), with a slightly higher value in January (2.33%). In this period, average temperatures ranged from 0.4 °C to 3.7 °C, accompanied by high atmospheric humidity (76.5–88.4%).

In spring, mortality showed markedly increased values, peaking in April (7.41%) and remaining similarly elevated in March (7.27%). In May, mortality decreased to a moderate level (5.52%), under an average temperature of 9.2 °C, precipitation of 57.7 mm, and a relative humidity of 72.1%. During the summer, mortality decreased in June (3.23%), peaked in July (6.15%), and declined slightly in August (4.09%). This period was characterized by high temperatures (28–29.6 °C) and lower humidity levels (55.1–62.1%).

In autumn, mortality remained relatively constant, ranging between 5.66% and 5.65%, against the background of moderate temperatures (14.3–16.6 °C) and a significant increase in precipitation (88.9 mm in September and 74.7 mm in October).

The data are summarized in Table 1, which highlights seasonal variations in mortality in relation to meteorological factors. The results suggest potential correlations between climatic conditions (temperature, humidity, and precipitation) and the frequency of porcine pleuropneumonia cases.

Analysis of humidity values shows that months with relatively lower levels, such as March (63.3%) and April (69.7%), coincided with the highest mortality rates (7.27% and 7.41%, respectively). In contrast, the lowest humidity was recorded during the summer months, particularly in July (51.4%), when mortality was lower (3.15%). These results indicate that the relationship between humidity and mortality varied across seasons, suggesting an interaction with other environmental parameters.

### 3.2. Data for 2022

The same epidemiological indicators were analyzed for the year 2022. The following section presents the monthly and seasonal distribution of mortality in relation to the meteorological parameters recorded during this period.

During the winter season, mortality ranged between 2.31% (January) and 2.91% (February), with a similar value recorded in December (2.57%). Temperatures were low (0.4–3.2 °C), while atmospheric humidity remained high (76–84.7%).

In spring, mortality reached the highest values of the year, peaking at 6.83% in March and 7.90% in April. In May, mortality remained at a high level (5.61%). This period was marked by a sharp increase in average temperature (from 1 °C in February to 8.7 °C in March), relatively low humidity (between 62.5% and 63.4%) and an increased amount of precipitation (up to 63.3 mm).

During the summer, mortality decreased compared to spring, being 4.41% in June, 3.59% in July, and 3.37% in August. High temperatures (23.2–29.1 °C) were associated with the lowest humidity values (54–59.8%), maintaining the overall trend of reduced mortality under thermal stress conditions.

In autumn, mortality increased again, with values of 6.30% in September, 5.52% in October, and 5.75% in November. This period was marked by moderate temperatures (10.7–14.5 °C) and relatively high humidity (67.2–85.0%), correlated with elevated precipitation levels (51.9–60.5 mm).

The data summarized in Table 1 indicate that, also in 2022, mortality associated with cases of porcine pleuropneumonia (APP) exhibited a clear seasonal variation, with peak values in spring and autumn and minimum levels during summer and winter.

### 3.3. Data for 2023

To highlight continuity and possible differences from previous years, the results corresponding to 2023 are presented below, capturing the same relationship between climatic parameters and herd mortality.

In the winter season, mortality ranged between 2.81% in February and 3.31% in December, with January showing a value of 3.29%. These months were characterized by low average temperatures (0.1–5 °C) and high relative humidity (79–89.6%).

During spring, the highest mortality rates of the year were observed, with 7.02% in March, 6.42% in April, and a maximum of 8.55% in May. In this period, temperatures were moderate (9.7–12 °C), while precipitation varied between 45.4 and 68.6 mm, and humidity between 61.7% and 73.7%.

In summer, mortality decreased to 3.23% in June, 3.83% in July, and 4.39% in August, corresponding to higher average temperatures (23–27.6 °C) and lower humidity levels (50.3–61.7%).

In autumn, intermediate mortality values were recorded, with 4.62% in September, 5.47% in October, and 4.76% in November. This season was marked by moderate temperatures (10.6–17 °C), high levels of precipitation (80.4–87.9 mm), and humidity ranging from 66.7% to 80.3%.

Overall, mortality in 2023 showed a seasonal distribution, with maximum values in spring (up to 8.55%), minimum values in winter and summer (2.81–3.83%), and intermediate levels in autumn (4.62–5.47%).

### 3.4. Data for 2024

**Figure 1:**
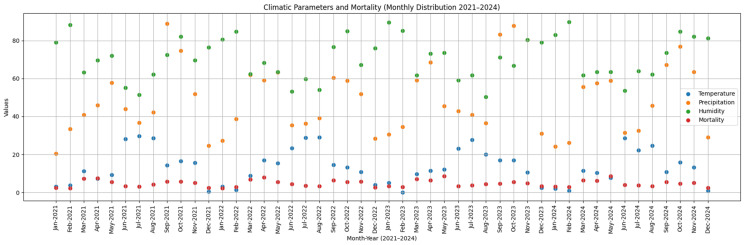
Monthly distribution of climatic parameters (average temperature, precipitation, and relative humidity) and associated mortality (%) recorded between 2021–2024. Each time point represents an individual month, resulting in a continuous sequence of 48 observations. The graphical representation highlights the variability of environmental conditions alongside fluctuations in mortality, allowing visual assessment of potential temporal associations and seasonal patterns.

The data collected throughout 2024 highlight significant seasonal variations in climatic parameters and their correlation with mortality caused by APP infections (Figure 1).

The cold season was characterized by low average temperatures (1–2.1 °C), high humidity levels (81.3–89.9%), and moderate precipitation (24.2–29 mm). Mortality ranged between 2.42% and 3.09%, representing the lowest values compared to other seasons. This suggests that low temperatures and high humidity may be associated with a lower frequency of severe outcomes.

Spring showed a progressive increase in temperature (7.7–11.4 °C) and precipitation (55.5–58.9 mm), concomitant with a decrease in humidity (61.8–63.4%). Mortality rose significantly, peaking at 8.67% in May, the highest annual value. This result indicates that the transition from cold to moderate temperatures, combined with decreasing relative humidity, may create favorable conditions for increased disease severity.

Summer was characterized by high temperatures (22.1–28.6 °C), low humidity levels (53.6–64%), and reduced precipitation (31.4–45.7 mm). Mortality remained relatively moderate, between 3.29% and 4.00%, suggesting that high temperatures may partially limit disease severity; however, heat stress and humidity fluctuations may contribute to sporadic cases.

Autumn presented moderate temperatures (10.8–15.8 °C), higher precipitation levels (67.2–76.9 mm), and increasing humidity (73.5–84.7%). Mortality reached intermediate values, ranging between 4.70% and 5.43%, indicating that this period represents a medium-risk season for the occurrence of outbreaks.

### 3.5. 2021–2024

**Figure 2:**
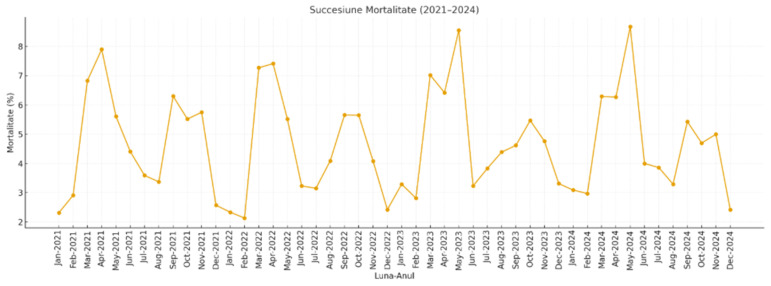
(2021–2024). Temporal succession of monthly mortality rates (%) in swine herds between 2021–2024. The graph illustrates the recurrent seasonal pattern, with pronounced peaks in spring (March–May) and autumn (September–November), corresponding to high-mortality periods consistently associated with respiratory lesions identified post-mortem. The visualization highlights both the cyclical nature of mortality dynamics and the inter-annual variability in magnitude.

The analysis of data collected between 2021 and 2024 revealed consistent seasonal variations in the mortality of pigs from the fattening sector, closely linked to fluctuations in climatic parameters (Figure 2). In all years examined, the spring months (March–May) recorded the highest mortality rates, peaking between 7.27% and 8.67%, associated with a fast transition from low to moderate temperatures, reduced relative humidity (between 61 and 64%) and increased precipitation—factors that contributed to microclimate instability and the development of thermal stress. In contrast, the summer season showed the lowest mortality rates (3.15–4.41%), despite high temperatures (>28 °C in June and July), suggesting an effective adaptation of the herds under adequate ventilation and hydration conditions, combined with moderate humidity and low thermal variability. Autumn exhibited intermediate mortality values (between 4.7 and 5.65%), reflecting the impact of transitional climate fluctuations on animal immunity. The cold season was consistently associated with the lowest mortality levels (2.13–3.31%), even under minimum temperatures close to 0 °C and high humidity (>85%), indicating effective protection provided by housing facilities. Correlation analysis suggests that mortality is influenced not by a single climatic parameter in isolation, but by the interaction of temperature, humidity, and precipitation, with effects amplified during seasonal transition periods.

Pearson correlation analysis revealed a statistically significant positive association between mortality attributed to APP infection and mean monthly temperature (r = 0.63, *p* < 0.01). A significant positive correlation was also observed between mortality and relative humidity (r = 0.54, *p* < 0.05). In contrast, no statistically significant correlation was detected between mortality and precipitation levels (r = 0.12, *p* = 0.41).

The multiple regression model yielded an R^2^ value of 0.45, indicating that approximately 45% of the variation in pig mortality can be explained by the combination of the three meteorological variables. Among these, temperature emerged as the strongest predictor (positive coefficient, *p* < 0.01), followed by humidity (moderate coefficient, *p* = 0.04), while precipitation did not contribute significantly to the model. The positive relationship between mean monthly temperature and pig mortality suggests that higher temperatures are associated with increased mortality rates, a trend further supported by the positive correlation identified in the statistical analysis.

The SARIMA (1,0,0) (1,0,0,12) model applied to the monthly pig mortality series revealed a stable seasonal component, with peaks consistently observed in April–May each year. Forecasts for the next six months (January–June 2025) indicate an increase in mortality from 3.13% in January to a peak of 9.44% in April, followed by a gradual decline during the summer months. These projections align with previous observations and can provide valuable guidance for planning preventive measures, such as optimizing ventilation, implementing pathogen-specific prophylactic protocols, and adjusting herd density according to the time of year. The predicted seasonal pattern underscores the potential utility of SARIMA models in supporting proactive management strategies in pig production.

The calculated Temperature–Humidity Index (THI) values showed marked seasonal variability during the study period (2021–2024). Overall, THI values ranged from 34.28 to 78.13, reflecting substantial differences between cold and warm seasons.

In 2021, THI values ranged from 37.34 in December to 78.03 in August, with the highest values recorded during the summer months (June–August), when average temperatures exceeded 28 °C. In 2022, THI values varied between 36.15 in February and 78.13 in July, again indicating pronounced summer heat conditions. Similarly, in 2023, THI ranged from 34.28 in February to 75.51 in July, reflecting lower winter temperatures and moderate summer humidity. In 2024, THI values ranged between 35.14 in February and 76.98 in June, with elevated values also observed during July and August. Overall, across the entire study period, the lowest THI values were recorded during winter months, while the highest values consistently occurred during summer.

The lowest THI values were recorded during winter months, particularly in February 2023 (THI = 34.28) and February 2024 (THI = 35.14), corresponding to low ambient temperatures combined with high relative humidity. Similar low THI levels were observed in December 2021 (35.99) and December 2024 (36.29).

Conversely, the highest THI values occurred during summer, when elevated temperatures coincided with moderate humidity levels. The maximum THI was recorded in July 2022 (THI = 78.13), followed by August 2021 (78.03), July 2021 (77.99), August 2022 (77.71), and June 2024 (76.98).

Intermediate THI values were typically observed during spring and autumn months, generally ranging between 45 and 65.

When analyzed annually, the mean THI values were relatively similar across the study years, with averages of 56.51 in 2021, 56.73 in 2022, 55.12 in 2023, and 54.22 in 2024, indicating moderate interannual variability.

Post-mortem examinations were conducted on approximately 90% of all deceased pigs. The overall mortality rate in the growing–finishing population was estimated at around 8% within a production system with an annual throughput of approximately 110,000 pigs. Gross lesions consistent with APP infection were identified in nearly 80% of the necropsied animals, suggesting a high prevalence of APP-associated pathology among mortality cases. Furthermore, pulmonary swab samples collected during diagnostic investigations were subjected to PCR analysis, with approximately 70% yielding positive results for APP. The isolates were further characterized, identifying serotype 7a as the predominant strain.

## 4. Discussion

The results obtained for 2021 highlight a clear seasonal pattern in mortality, with variations associated with climatic conditions, with higher rates recorded in spring and autumn, while lower values were observed in winter and summer. These findings suggest that fluctuations in temperature and humidity may act as stress factors for pigs, facilitating the occurrence of respiratory disease episodes. In 2022, the seasonal distribution of mortality confirmed the trends observed in the previous year, with peak values again in spring and autumn. However, the amplitudes were more pronounced, particularly in April, compared to 2021. This indicates that inter-annual variability in climatic conditions, such as abrupt temperature shifts combined with fluctuations in humidity, may further exacerbate disease dynamics. The consistency of these patterns across consecutive years reinforces the hypothesis that environmental stressors significantly contribute to the epidemiology of respiratory diseases in swine populations. In 2023, this seasonal pattern was again confirmed, with mortality reaching its highest values in spring, remaining low in winter and summer, and stabilizing at intermediate levels in autumn, thus further strengthening the evidence that climatic variability is associated with variations in mortality. Consistent with earlier observations, the 2024 analysis revealed a significant mortality distribution across seasons in affected herds. Mortality peaked in spring, reaching its maximum in May, when moderate temperatures and reduced humidity likely favored the progression of respiratory conditions. Autumn displayed intermediate values, indicating that climatic transitions played a decisive role in shaping respiratory disease dynamics during this year.

Spring months, particularly the transition from cold to warm seasons, were associated with the highest mortality rates. Although temperatures and humidity remained within moderate ranges, the abrupt seasonal changes likely acted as major stress factors, predisposing animals to respiratory or digestive disorders. Additional influences, such as dietary adjustments and adaptation to changing housing microclimates, may also have contributed to the increased susceptibility observed during this period. During summer, mortality reached its lowest levels, despite high ambient temperatures. This suggests a good adaptation of pigs to heat stress when adequate ventilation and hydration are provided. The relatively low humidity and stable thermal conditions likely contributed to maintaining animal comfort and reducing disease incidence. Mortality rates increased slightly during autumn, notably toward the end of the season, reflecting the effects of seasonal transitions on animal health. Fluctuations in temperature and humidity during this period may weaken the pigs’ immune response, increasing their susceptibility to disease. In winter, although average temperatures were low, mortality also remained reduced, indicating that appropriate housing conditions offered effective protection against cold stress. High humidity during this season did not appear to have a major negative effect under the conditions analyzed.

The seasonal distribution of mortality showed higher average values during climatic transition periods—spring and autumn, compared with summer and winter. This trend supports the hypothesis that pronounced variability in the microclimate within housing facilities, combined with frequent ventilation adjustments and a possible reduction in nonspecific resistance, may promote the onset and exacerbation of respiratory diseases in finishing pigs. During periods with a high Temperature–Humidity Index (THI), the combination of temperature and humidity increases heat stress and mortality, including in the context of transport and slaughter, which may explain the peaks observed in spring and autumn [18,19].

Seasonal fluctuations in pig mortality support the hypothesis that microclimatic instability and heat stress play a crucial role in exacerbating respiratory diseases during transitional months. Given swine’s limited sweating capacity [20], sudden changes in temperature and humidity increase pigs’ susceptibility to disease during spring and autumn.

The Pearson correlation coefficient analysis revealed a significant positive association between mortality and average monthly temperature as well as between mortality and humidity. In contrast, no statistically significant relationship was observed between mortality and precipitation amount. These findings suggest that climatic variables are associated with variations in mortality and that a climate characterized by high temperatures and increased humidity may amplify heat stress and create favorable conditions for the proliferation of respiratory pathogens, such as APP. These results are supported by a study which demonstrated that, under conditions of 80% humidity, each degree above the critical threshold (approximately 22–25 °C) reduces daily feed intake and affects both respiratory rate and body temperature [21], indicating a decrease in thermal comfort and an increase in the animals’ vulnerability to respiratory diseases. Therefore, these environmental factors may represent important determinants of increased mortality in finishing pigs.

Molecular monitoring was performed periodically using PCR testing, with an average frequency of every 4–6 months. Samples consisted of pulmonary swabs, of which approximately 70% tested positive for APP. Antimicrobial susceptibility testing was also performed at two-year intervals at a private veterinary laboratory (Bucharest, Romania), revealing changes in resistance patterns over the course of the study period. The proportion of positive cases for APP varied across seasons, with higher detection rates observed during periods characterized by increased humidity and temperature fluctuations. Clinically, respiratory signs were more frequently observed during these periods, affecting a higher proportion of animals within the batches. Necropsies were performed on approximately 90% of deceased animals, and lung lesions consistent with pleuropneumonia were frequently identified in these cases. Among finishing pigs, approximately 80% of mortality cases were attributed to APP-associated disease, while the remaining 20% were linked to other conditions, including hernias, ulcerative vegetative endocarditis, and polyserositis. These findings suggest a potential association between climatic conditions and both the occurrence and severity of APP infections, beyond mortality data alone.

Beyond environmental influences, pathological findings further elucidate the multifactorial etiology of the observed mortality patterns. Post-mortem examinations revealing extensive pneumonia—unilateral or bilateral—with diffuse hemorrhages, friable necrotic regions, and fibrinous pleuritis closely resemble those observed in enzootic pneumonia caused by *Mycoplasma hyopneumoniae*, one of the primary agents in the Porcine Respiratory Disease Complex (PRDC) [22]. Moreover, the involvement of other PRDC pathogens should be considered a major contributor to lesion severity. Co-infections with agents like APP contribute to more severe and multifocal lesions, particularly the necrotic and fibrinous changes often observed in advanced pneumonia. Field necropsy data suggest that the severity of lesions often reflects synergistic pathogenic interactions rather than a single etiological agent [23].

PRDC is characterized by pneumonias with highly variable pathological presentations, encompassing both aerogenous and hematogenous forms and a frequently multimicrobial etiology. It is widely recognized as a multifactorial condition resulting primarily from the interaction between bacterial and viral pathogens, and less commonly involving parasitic agents, all of which are further modulated by environmental and management-related stressors as well as pig-specific factors [24]. In this context, seasonal increases in mortality, particularly during spring, may reflect a period of heightened susceptibility, when animals are more vulnerable to respiratory pathogens and the effects of co-infections become more pronounced.

Climatic conditions, particularly during periods of low outdoor temperature, can influence the indoor environment through the performance of heating and ventilation systems, thereby contributing to the environmental modulation of respiratory disease dynamics. When these systems are not properly balanced, increases in relative humidity and carbon dioxide concentration may occur, potentially contributing to unfavorable conditions within animal housing. It has been suggested that climatic factors may play a more important role in the spread of less contagious respiratory diseases, such as those caused by APP, compared to other contagious pathogens like *Mycoplasma hyopneumoniae*. Overall, pig productivity is likely influenced by the combined effect of infections and environmental stressors to which animals are exposed [25].

The integration of the temperature–humidity index (THI), as a composite indicator of thermal stress, provides additional insight into the relationship between climatic conditions and mortality dynamics observed in this study. Across all analyzed years, THI values exhibited a clear seasonal pattern, with low levels during winter, moderate values in spring and autumn and elevated levels in summer. Notably, the highest mortality rates were not associated with peak THI values recorded during summer, but rather with intermediate THI ranges characteristic of spring and autumn. This suggests that, in the studied herd, abrupt fluctuations and transitions in THI, rather than sustained heat stress, may represent a more critical factor influencing disease expression. Moderate THI levels, combined with rapid changes in temperature and humidity, likely contribute to microclimatic instability within housing facilities, impairing respiratory defense mechanisms and increasing susceptibility to respiratory diseases. In contrast, during summer, despite high THI values indicative of potential heat stress, mortality remained comparatively lower, possibly reflecting physiological adaptation and effective environmental management. These findings support the hypothesis that transitional thermal stress, reflected by dynamic changes in THI, plays a more significant role in exacerbating mortality associated with respiratory diseases than constant exposure to high temperature–humidity conditions. The interpretation of the THI in relation to animal health should also consider established threshold classifications reported in the literature. One study categorized THI values into biologically relevant ranges, including danger due to cold (<59), lower critical conditions (59–60), suboptimal conditions (61–62), thermoneutral or safe conditions (63–70), above recommended levels (71–72), upper critical heat stress (73–75), and danger due to heat (>75) [26]. In the context of the present study, although the calculated THI values are not directly comparable in absolute terms due to differences in the applied formula, the observed seasonal dynamics—especially during spring and autumn—may reflect similar deviations from the thermoneutral zone. This supports the hypothesis that not only extreme thermal stress, but also suboptimal or fluctuating microclimatic conditions, can negatively influence respiratory health and contribute to increased respiratory disease-related mortality in finishing pigs.

This study presents several limitations that should be considered when interpreting the results. First, all data were derived from a single commercial farm, which may limit the generalizability of the findings to other production systems with different management practices, housing conditions, or health status. Second, although mortality was analyzed in the context of respiratory disease dynamics, confirmatory diagnostic testing was not systematically performed for all affected individuals, which restricts the ability to attribute outcomes to specific etiological agents. Therefore, the observed mortality should be interpreted as being associated with respiratory conditions rather than a single pathogen. Third, the study relied on external meteorological data, and detailed information regarding the internal microclimate of the facilities was not available. As a result, potential discrepancies between outdoor environmental conditions and the actual microclimatic exposure of animals may have influenced the observed associations. Despite these limitations, the study provides valuable insights into the relationship between climatic variables and variations in mortality, highlighting the importance of environmental management in swine production systems.

## 5. Conclusions

Seasonal fluctuations in climatic conditions play a critical role in shaping mortality patterns associated with porcine pleuropneumonia during the fattening period. Mortality peaked in spring, associated with rapid temperature increases, decreased humidity, and microclimatic instability, which likely enhanced thermal stress and susceptibility to infection. Winter showed low mortality rates, highlighting the protective role of stable housing and controlled microclimate.

Environmental factors can interact with respiratory pathogens, amplifying disease severity during transitional periods. These findings emphasize the importance of microclimate management and season-specific preventive strategies to improve herd health and productivity.

The results provide a basis for developing predictive algorithms that correlate local climatic data with APP outbreak risk, potentially transforming health surveillance in commercial swine farms. Given the economic impact of APP on swine health, these findings can support the implementation of seasonally tailored preventive strategies, including vaccination and therapeutic interventions guided by climatic forecasts.

## Figures and Tables

**Table 1:** Monthly mortality (%) in the fattening sector and corresponding meteorological parameters (average temperature, precipitation, and relative humidity) recorded during 2021–2024. Data are presented by month and season, illustrating seasonal variations in mortality associated with cases episodes of porcine pleuropneumonia.

Year	Month	Average Temperature (°C)	Precipitation (mm)	Humidity (%)	Mortality (%)
2021	January	3.2	20.5	79.1	2.33
	February	3.7	33.5	88.4	2.13
	March	11.2	40.9	63.3	7.27
	April	7.3	46.0	69.7	7.41
	May	9.2	57.7	72.1	5.52
	June	28.1	44.0	55.1	3.23
	July	29.6	36.7	51.4	3.15
	August	28.5	42.1	62.1	4.09
	September	14.3	88.9	72.6	5.66
	October	16.6	74.7	82.2	5.65
	November	15.6	51.8	69.6	5.08
	December	0.4	24.7	76.5	2.42
2022	January	3.2	27.3	80.6	2.31
	February	1.3	38.7	84.7	2.91
	March	8.7	61.9	62.5	6.83
	April	16.9	59.2	68.4	7.90
	May	15.4	63.3	63.4	5.61
	June	23.2	35.4	53.2	4.41
	July	28.8	36.3	59.8	3.59
	August	29.1	39.2	54.0	3.37
	September	14.5	60.5	76.7	6.30
	October	13.2	58.8	85.0	5.52
	November	10.7	51.9	67.2	5.75
	December	4.0	28.4	76.0	2.57
2023	January	5.0	30.6	89.6	3.29
	February	0.1	34.4	85.2	2.81
	March	9.7	59.2	61.7	7.02
	April	11.5	68.6	73.1	6.42
	May	12.0	45.4	73.7	8.55
	June	23.0	42.8	59.1	3.23
	July	27.6	40.8	61.7	3.83
	August	20.0	36.5	50.3	4.39
	September	17.0	83.3	71.2	4.62
	October	17.0	87.9	66.7	5.47
	November	10.6	80.4	80.3	4.76
	December	2.4	31.0	79.0	3.31
2024	January	2.1	24.2	83.1	3.09
	February	1.0	26.2	89.9	2.97
	March	11.4	55.5	61.8	6.29
	April	10.4	57.6	63.5	6.27
	May	7.7	58.9	63.4	8.67
	June	28.6	31.4	53.6	4.00
	July	22.1	32.6	64.0	3.86
	August	24.7	45.7	62.1	3.29
	September	10.8	67.2	73.5	5.43
	October	15.8	76.9	84.7	4.70
	November	13.2	63.6	82.2	5.00
	December	1.0	29.0	81.3	2.42

## Data Availability

All data generated or analyzed during this study are included in this article.
